# Caloric Stimulation with Water and Air: Responses by Age and Gender

**DOI:** 10.22038/ijorl.2020.49305.2632

**Published:** 2021-03

**Authors:** Lilian Felipe, Rocio Cavazos

**Affiliations:** 1 *Department of Speech and Hearing, Lamar University, Beaumont, TX. USA. *; 2 *luminense Federal University, Niteroi, RJ. Brazil.*

**Keywords:** Air, Age, Caloric test, Nystagmus, Water

## Abstract

**Introduction:**

The caloric test is a well-known valuable clinical instrument that can evaluate and quantify the functional status of both lateral semicircular canals. The American National Standard Institute (ANSI) does not include air as a standard method for caloric stimulation due the lack of published data to determine response variability comparable to water. Due the controversy about air irrigators, it is worthwhile to evaluate the presence of differences between the two irrigation methods in caloric response. The goal is to compare, by age group, the post caloric responses with water and air according gender and age.

**Materials and Methods:**

Individuals without otoneurologic complaints were selected and divided in groups. All were submitted to caloric bithermal stimulation with water at temperatures of 44°C and 30°C (Micromedical Technologies, Inc., USA) and air at temperatures of 50°C and 24°C (Micromedical Technologies, Inc., USA).

**Results:**

91 subjects were evaluated (46 men and 45 women) with a mean age of 43 years old. The caloric response was similar between genders (P=0,958) and no statistical difference was observed comparing both stimulus (P=0,93). It was identified that the Slow-Phase Velocity (SPV) was lower for the group older than 60 years comparing to the other groups.

**Conclusion:**

For the caloric test, the stimulus with air was confirmed as similar as stimulation with water, including absolute values. Lower values for SPV were found for elderly population.

## Introduction

The caloric test allows comparing the function of each vestibular labyrinth (semicircular lateral canal), defining which side is compromised (1). It is performed to investigate lateralized peripheral lesions by comparing the nystagmus responses between the right and left labyrinths. The method comprises timed bithermal stimulation with water or air of the external auditory canal, generating a temperature gradient that moves across the ear toward the horizontal canal on the side of the irrigation. A warm stimulus causes endolymph within the horizontal canal to rise, which creates ampulopetal endolymph flow (excitatory neural response); and cool stimulus increases the endolymph density resulting in ampulofugal endolymph flow (inhibitory response) (2).

The objective of the caloric test is to detect the level to which the vestibular system reacts to the stimulus and to define if the reaction in each side is symmetric or asymmetric. It is an assessment of the lateral semicircular canals exclusively – it does not evaluate vertical canals or otolith organs (2,3).

The stimulus used to induce nystagmus response in the caloric test are water and air (2,4). Water stimulation was described by Barany (1906) (5) and its standard protocol was established by Fiztgerald and Hallpike (1942) (6). The external auditory canal is irrigated with 250 milliliters (ml) of distilled water for 40 seconds at temperatures of 44°C and 30°C, leading to an endolymphatic current in the lateral semicircular canal on either side, with a frequency around 0.003 Hertz (Hz) (5,6). In air stimulation, most of the studies presented a protocol with an air-flow of 08 L/min at temperatures of 50°C and 24°C for 60 seconds (7-9), generating an endolymphatic current similar to that generated with water at temperatures of 44°C and 30°C (2). Other stimulus patterns have been described: temperatures of 45.5°C and 27.5°C for 100 seconds with air flow of 13 L/min or temperatures of 18°C and 42°C for 80 seconds with air flow from 7 to 8 L/min (10).

Thus, unlike the water stimulus, in the air stimulus, the protocols vary in relation to the equivalent temperatures for water/air stimulus (7,33,^10^-^12^). Water stimulation is considered the reference of objective measurement of caloric response for evaluation of peripheral vestibular function (1). However, caloric testing with water is more inconvenient for the patient and provokes more intense neurovegetative reactions when compared to air stimulation (13). Therefore, several services have adopted the technique of air stimulation as a standard for caloric testing, notwithstanding this technique have been recommended to be used just in special cases, as tympanic membrane perforation (4). 

There is debate about the clinical use of air irrigators. The American National Standard Institute (ANSI) does not include air as a standard method for caloric stimulation due the lack of published data to determine response variability comparable to water (ANSI 2009). Due the controversy about air irrigators, it is worthwhile to evaluate the presence of differences between the two irrigation methods in caloric response (14).

The caloric test is analyzed by comparative evaluation of the peak slow component eye velocity for all irrigations. The four values are applied to produce a percent response level to compare both sides (right and left), called Unilateral Weakness (UW), and also to determine the directional preference of eye movement, denominated Directional Preponderance (DP). The Slow Phase Eye Velocity (SPV) values of each stimulation should be taken from the range of prominent magnitude on the responses (2,3).

The significance of this study is interconnected to controversies that remain in scientific literature about: 1) the normative values for air comparing with water in the same sample, since few studies assess caloric comparing these two methods (14); 2) the difference in responses between which authors concluded that water produced a stronger response comparing to air and the use of air stimulation in clinic evaluation (13).

The objective of this study was to compare the caloric response between water / air stimuli and by age and gender among individuals without otoneurological complaints. 

## Materials and Methods:

The procedures followed were in accordance with the ethical standards of the responsible Institutional Review Board (IRB-FY18-309) and with the Declaration of Helsinki, written informed consent was obtained following a detailed explanation of the procedures. 

This was a cross-sectional study. All individuals in this study had normal hearing thresholds verified by air and bone conduction pure tone audiometry bilaterally and normal middle ear function as demonstrated by the immittanciometry. None of them had any past or current presence of otological symptoms. Neither had any neurological or neuromuscular condition. All participants had no complaints about corporal balance, no history of cervical spine injury, and no antecedents of exposure to excessively loud sounds. All of them were apprised concerning the study and a written consent form was taken before submitted to caloric test with water and air stimulations.

Between exams, the minimum period of one week was respected, in order to avoid interference between the results. All subjects underwent to a previous otorhinolaryngological evaluation. Participants followed the guidelines adopted before the exam, as follows: suspension of non-essential drugs and alcoholic beverages for 72 hours, suspension of smoking for 24 hours and do not ingest food three hours before the exam. 

Prior to the stimulation, the ears were inspected for the presence of cerumen and all oculomotor movements were evaluated. Abnormality in any of the movements was considered an exclusion criterion for participation in the study.

The subjects were instructed on each of the stages of caloric testing. Both stimulations were performed by the same examiner, who maintained the systematization for all stages of the test. The position adopted for the caloric test was supine with head inclined at 30º, placing the horizontal canals in a near vertical position (11,15). 

During the caloric stimulation with water, the external auditory canal was stimulated for 40 seconds with 250 ml of water at the temperatures of 44°C and 30°C (Micromedical Technologies, Inc., USA). For the caloric test with air, an airflow was presented for 60 seconds, with flow of 8L/min, at temperatures of 50°C and 24°C (Micromedical Technologies, Inc., USA). The air stimulator used in this study contains a coupled otoscope, allowing the vision of the tympanic membrane, to facilitate deeper irrigation in the external auditory canal, considered essential for correct stimulation. It was allowed at least seven minutes between the start of one stimulation and the start of the next irrigation to guarantee that the response of one irrigation does not influence the subsequent (16). The examiner used mental tasks to keep the individual alert during the test (17). 

The caloric response was calculated regarding the peak of Slow Phase Velocity (SPV) of the nystagmus in degrees per second (2-10). The four responses were represented by the subsequent codes: WR − warm stimulus in right ear; WL − warm stimulus in left ear; CR − cool stimulus in right ear; CL − cool stimulus in left ear (18,19). Unilateral Weakness (UW) and Directional Preponderance (PD) were characterized by the follow formulas (18). 

Unilateral Weakness (%) = (WR+CR) – (WL+CL)/ (WR+ WL+CR+CL) x 100

Directional Preponderance (%) = (WR+CL) – (WL+CR)/ (WR+ WL+CR+CL) x 100

Statistical Analysis

The data generated by this study was analyzed using the SPSS Statistical Program, SPSS Inc., IBM, Chicago, USA. Post-caloric responses were evaluated in relation to gender, stimulus type, and age. 

In the statistical comparison, the difference between the means was compared using the T test, using the level of statistical significance of 5%. The t-test was adequate for the comparison of the groups since they are independent samples in which the studied variables are quantitative and have normal distribution in both groups with the same standard deviation. The normality of the continuous variables was verified by the Kolmogorov-Smirnov test and the homoscedasticity between the groups by the Levene’s test.

## Results

Ninety-one subjects (182 ears) were evaluated (46 men and 45 women) with a mean age of 43 years, ranging from 20 to 66, with a standard deviation of 15. 

The comparison of caloric response in relation to gender presented no statistically significant difference (P=0.958). Comparing the SPV values obtained in each ear with water and air stimulation, no difference was observed in relation to the stimulation side (Table.1).

**Table 1:** Slow Phase Velocity (SPV) comparison of air versus water stimulation for right and left. N = 91 subjects. XXX 2019

	**WATER**			*P-value*	**AIR**			*P-value*
	Median	Min-Max	SD		Median	Min-Max	SD	
Right Ear	16.18	4-44	7.43	0.43	15.36	4-46	8.33	0.99
Left Ear	16.12	4-50	7.89		15.3	3-46	8.33	

Legend: P = significance level – T-test; SD = standard deviation; min = minimum; max = maximum

Thus, SPV was similar between ears and gender, with water and air stimulation. Therefore, comparative analyzes regarding stimulation type and age range were performed independently of the stimulation side and gender. The SPV values for air and water stimulation according to age range are shown in Table 2. 

**Table 2:** Slow Phase Velocity (SPV) values for water and air stimulation in relation to age. N=91. XXXXX. 2019

**Age Group**	**N**	**WATER**			**AIR**			
		**Median**	**Min-Max**	**DP**	**Median**	**Min-Max**	**DP**	**P**
20-30	26	17.49	4-47	8.20	16.73	3-46	7.89	0.45
31-40	15	20.25	10-50	8.52	18.01	4-45	8.60	0.68
41-50	19	14.33	4-37	7.14	15.86	4-37	8.57	0.57
51-60	18	15.26	7-29	6.04	14.40	7-29	8.06	0.61
>60	13	12.44	4-26	5.36	9.31	3-20	4,26	0.23
Total	91	16,12	4-50	7.67	15.25	3-46	8.20	0.93

Legend: P = significance level – T-test; SD = standard deviation; min = minimum; max = maximum.

*Statistically significant values (P<=0.05). T-test

**Fig 1:**
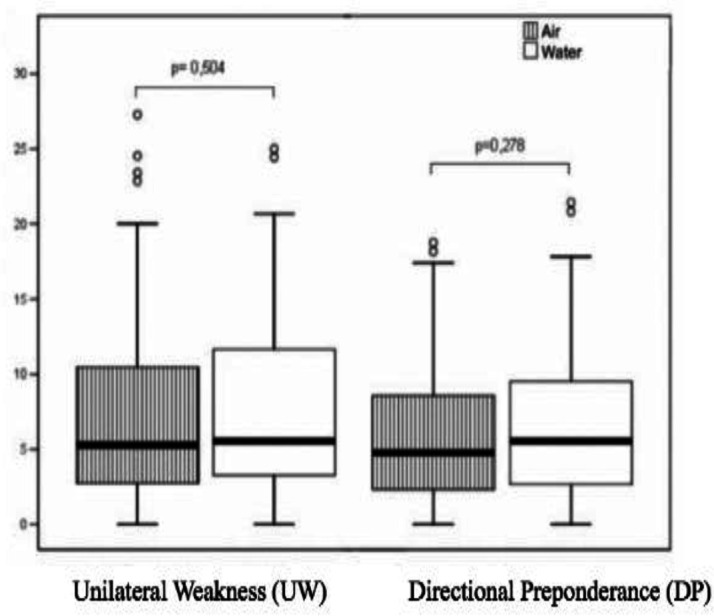
Comparison of mean UW and DP values in relation to air and water caloric stimulation. N=91. XXXX. 2019. Legend: P= significance level- T-test (<=0.005)

The relative values, UW and DP, were similar between both types of stimulation for both UW (P=0.504) for DP (P=0.278). The results are described in Figure 1.

**Fig 2:**
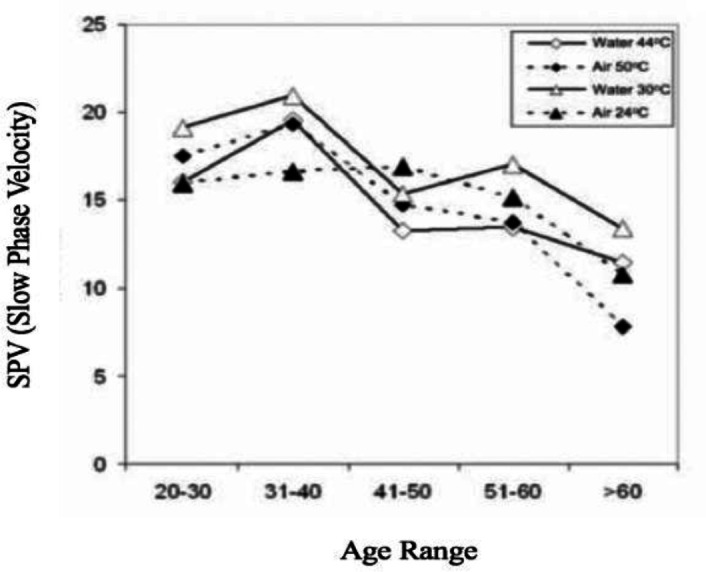
Slow Phase Velocity values in relation to air and water caloric stimulations. N=91. XXXX. 2019

Comparison of SPV values with air and water stimulation in relation to age group is represented in the Figure 2. It was observed that the SPV was lower for individuals in the group aged over 60 years compared to the other groups. 

This difference was statistically significant when comparing the SPV values between the 20-30-year-old group and subjects in the group above 60 years old (Table.3).

**Table 3:** Slow Phase Velocity (SPV) values for water and air stimulation in relation to age. N=91. XXXXX. 2019

	**Age Group**	**Median**	**Min-Max**	**SD**	**P**
Water	20-30	17.49	4-47	8.20	0.007
	>60	12.44	4-26	5.30	
Air	20-30	16.73	3-46	8.20	0.004
	>60	9.30	3-20	4.20	

Legend: P = significance level – T-test; SD = standard deviation; min = minimum; max = maximum. *Statistically significant values (P<=0.05). T-test

## Discussion

The caloric test is part of the assessment of patients with dizziness, as it provides a measure of vestibular deficit. The fundamental of caloric stimulation is that normal system tends to symmetrical and measurable caloric response within a previously recognized range of normality. Relevant aspect is to establish similarity between temperature variations for the stimulus with water or air in the caloric test (7,11,18). For the air stimulus, several parameters have been tested, with equivalence temperatures with the water stimulus ranging from 42ºC to 54ºC for the warm stimulus and 18ºC to 24ºC for the cold stimulus. 

It was found that the air stimulation protocol used in this study (24ºC and 50ºC) was able to produce similar responses to the standard water caloric testing protocol in a group of normal individuals (7,11,12,21). The results comparing the air and water stimulation revealed no statistically significant differences of SPV values in relation to the ears (Table 1), which is consistent with studies in which the laterality variable was evaluated in the caloric test (11,20). The parameters used as normal limit in the comparative analysis of UW and DP vary according to the services (8-12,22,24-26). Regarding the stimulus with water, the literature presents confidence limits for UW=33% and DP=22% (10). SPV values between 1º/s and 35.8º/s and confidence limits for UW=23% and DP=28% (23). In the present study, the maximum value for water stimulated in UW result was 25% and for DP 21%. For air stimulation, the maximum values for UW and DP were, respectively, 27% and 18% (Table.2 and Fig. 1).

 These findings supported the noteworthy importance of internal validation of the results in each service to increase the credibility and confidentiality of the test. Comparing the SPV values by age group, it was observed that the values from individuals older than 60 years were statistically significant comparing with the group of 20-30 years, which demonstrates a lower vestibular response to caloric stimulation in older individuals. Decreased response to caloric testing with increasing age has been described in the literature and can be correlated to the aging of the vestibular system (9, 24-26). Elderly may present a decreased caloric response in relation to the absolute values, but not varying the UW or DP (24). Several clinics around the world have been choosing the air equipment besides the water due the advantage of air stimulation eliminate the necessity of water collection and possibility of examination in subjects with tympanic membrane perforation (22). Although it is important to reinforce that this technique is useful in particular situations, suggesting that a good clinical laboratory should have both types of stimuli for the caloric test. As known, considering both stimuli, the air stimulation requires more technical skills than water. Changes in air stimulation depth may cause variations in caloric response, with a reduction of around 20% to 40% (12). Therefore, the air stimulation demands more experience from the examiner when compared to water stimulation (10,12). The use of the air stimulator with coupled otoscope was important to ensure the same depth of insertion of the stimulator in the external auditory canal, ensuring the quality of the caloric response (7).Additionally, in the temperature of 50°C with air, the SPV values were significantly higher than other temperatures in some subjects of this sample, with some of them relating pain (sensation of burning) during the stimulation. Studies with air stimulation at this temperature report some subjects having superior responses when compared to the values found in water stimulation due to the discomfort caused by hot air in the auditory external canal (12), that can lead to an incorrect final conclusion for a non-experience examiner. 

**Future Directions**

It is essential to mention that new methods with the current advance in technology and instruments have offered to verify the vestibular function and presence of abnormalities. These comprehend the video Head-Impulse Test (vHIT) that evaluates all semicircular canals and the ocular and cervical Vestibular Evoked Myogenic Potential (VEMP), presenting data about the otolith organs (7,15). 

The caloric test is characterized by unilateral stimulation in low frequency (influenced by visual information) while the vHIT, the stimulation is bilateral and in high velocities (necessary to isolate the vestibular reflex) (24). 

It is important to recognize that the caloric test has resisted the test of time and all these tests are complementary to each other in the otoneurological evaluation and management of the patient with dizziness. 

## Conclusion

In individuals without otoneurological complaints, the caloric test with air generated similar SPV responses to those generated with water. It should be noted, however, that air stimulation requires greater technical skill from the examiner. Ensuring this issue and focusing on patient comfort, air caloric stimulation showed excellent agreement with water stimulation and can be used instead. The values for UW was 25% and 27% and DP with 21% and 18% for water and air, respectively. 

Lower values with statistical significance for SPV were found for elderly population comparing both stimulation types.
